# Post marketing surveillance of selected veterinary medicines in Tanzania mainland

**DOI:** 10.1186/s12917-022-03329-x

**Published:** 2022-06-09

**Authors:** Adam M. Fimbo, Betty A. Maganda, Kissa W. Mwamwitwa, Itikija E. Mwanga, Engelbert B. Mbekenga, Seth Kisenge, Sophia A. Mziray, Gerald S. Kulwa, Yonah H. Mwalwisi, Danstan H. Shewiyo

**Affiliations:** 1 Tanzania Medicines and Medical Devices Authority, P.O BOX 77150, Dar es salaam, Tanzania; 2 Department of Pharmaceutics and Pharmacy Practice, Muhimbili University of Health and Allied Sciences, P.O BOX 65013, Dar es salaam, Tanzania

**Keywords:** Falsified, Post-marketing surveillance, Product Information Review, Substandard, Screening, Veterinary medicines

## Abstract

**Background:**

Veterinary medicines have been widely used for the prevention and treatment of animal diseases. Globally, the veterinary medicine industry is growing. However, there is a significant increase of concern on the quality of veterinary medicines in various developing countries’ legal markets. Poor-quality medicines are associated with treatment failure, development of drug resistance, increased healthcare cost, and death. These reasons warrant a need for monitoring the quality of the medicines circulating in the Tanzania Mainland.

**Methods:**

This was a survey study and veterinary medicines samples were collected from 9 out of 26 regions of Tanzania mainland between 2014 and 2017. Veterinary medicines were sampled from wholesale pharmacies, retail pharmacies, veterinary clinics and Veterinary Accredited Drug Dispensing Outlets (ADDO-vet). All sampled medicines were subjected to product information review and full quality control testing at the Tanzania Medicines and Medical Devices Authority-World Health Organization prequalified laboratory.

**Results:**

A total of 238 samples of veterinary medicines were collected. Out of these, 97.1% (231/238) were subjected to full quality control testing and product information review. All sampled veterinary medicines conformed to visual appearance, clarity, pH, solubility and sterility tests. Also, of the sampled veterinary medicines 97.8% (226/231) and 89.2% (206/231) passed identification and assay tests, respectively. As well as, the majority of the collected samples 92% (219/238) failed to comply with product information requirements. The most observed deficiencies on product information were inadequate information on the package insert 94.1% (224/238), inappropriate storage conditions 55.5% (132/238) and lack of Tanzania registration number 27% (64/238).

**Conclusion:**

Veterinary medicines with poor quality were found circulating in the legal markets of Tanzania. This can potentiate treatment failure and the development of drug resistance in animals and humans. Post marketing surveillance program will continue to be implemented to ensure that only good quality, safe and efficacious medicines are circulating in the Tanzania Mainland market.

## Background

Animal diseases are of considerable importance to human welfare, as a cause of illness in man and as a factor in interrupting the food chain [1, 2]. Animals contribute approximately 60 to 75% of all emerging human infectious diseases [3]. Veterinary medicines have been used in the treatment, control, and prevention of animal diseases that affect the health of companion, domestic, exotic animals, and wildlife [4, 5]. The quality of veterinary medicines throughout the shelf life determines the effectiveness in overcoming animal diseases through treatment, prevention and control [6]. National medicine regulatory authorities have the responsibility of regulating the quality of medicines circulating in their market thus, preventing availability of falsified and substandard medicines on the market [6–8].

The problem of poor-quality medicines is persistent in various Sub-Saharan African (SSA) countries, of which veterinary medicines are of no exception [5, 9–18]. Reports from various SSA countries have shown that markets of these countries are flooded with veterinary medicines of doubtful quality [5, 13–18]. It is estimated that about 50% of veterinary medicines sold in parts of Africa and Asia are substandard and/or falsified [9]. Falsified and substandard medicines may lead to drug toxicity, development of drug resistance, treatment failure, increased treatment costs, and deaths. The same state of affairs relates to veterinary medicines [14, 19, 20]. Approximately, 30%, 55%, and 22% of veterinary medicines, circulating in Kenya, Nigeria, and South Korea respectively, have been reported to be substandard [5, 13, 16]. Not only that but also, in another study conducted in Kenya, the majority of veterinary medicines circulating in the legal market were found to have low content (0–85.4%) and few had high content (118–120.6%) of the claimed active principle ingredient (API) [15]. The Cameroon and Senegal studies as well indicated that veterinary medicines circulating on their markets were of poor quality [17, 18]. The quality of medicines can be compromised during the manufacturing process, distribution and storage due to high temperature and humidity. Therefore, safeguarding animals and human health through monitoring of the quality of veterinary medicinal products is of clinical importance as it ensures quality and efficacy whilst preventing the development of drug-resistant strains, treatment failure, and wastage of resources [9]. This study evaluated the quality of selected veterinary medicines circulating in the Tanzania Mainland Market. The primary objective of this study was to determine the proportion of poor quality veterinary medicines circulating on the Tanzania Mainland market and the secondary was to determine the quality of information on the primary and secondary packaging and availability of package information leaflets.

## Methods

### Study design

This was a survey cross-sectional study as part of the ongoing Post Marketing Surveillance (PMS) Program designed to assess the quality of registered human and veterinary medicines in Tanzania Mainland.

### Study settings and duration

This study was conducted in 9 regions of Tanzania's mainland of which are Arusha, Dar es Salaam, Mara, Mbeya, Mwanza, Shinyanga, Dodoma, Manyara and Kilimanjaro. Selection of the regions was based on the following criteria: regions bordering other countries, areas reported to have veterinary medicine of poor quality, regions not involved in the previous PMS program or highly pastoralists’ regions. Samples were collected from veterinary medicines distribution outlets between 2014 and 2017.

### Veterinary medicines sampling sites and sample collection

Convenience sampling method was used for the collection of the selected veterinary medicines.

The inspectors collected the samples using routine regulatory procedures. The focus was on both imported and locally manufactured veterinary products.

Collection sample sites were selected in such a way to cover urban, sub-urban, and rural areas and all distribution outlets [21]. Veterinary medicines were sampled from retail pharmacies, wholesale pharmacies, veterinary clinics, and veterinary Accredited Drug Distribution Outlets (ADDO-vet). The selection of outlets per region was based on a systematic random sampling method.

Sample collectors were trained drug inspectors who executed the work according to the pre-prepared sampling plan and Tanzania Medicines and Medical Devices Authority (TMDA) standard operating procedures. At each outlet the targeted product, dosage form, strength and pack, two batches per product and one batch per brand were sampled. For water-soluble powder 10 sachets, solutions of 100 mL 15 bottles and injectables 40 vials were collected.

All samples were collected in their original containers and detailed information was filled in the Sample Information Collection Form. Each collected sample was coded for traceability. Sample code included brand name, region, sampling site and sampling date. The following information was recorded for each sample in the Sample Information Collection Form: Brand and generic names, dosage form, strength, batch or lot number, date of manufacturing and expiration, name and address of the manufacturer, country of origin, TMDA registration number, packaging and pack size, availability of package information leaflet, language and storage instructions, physical appearance of the primary and secondary package, site and date of sample collection [21].

Coded samples with their respective Sample Information Collection Form were kept in the labelled sampling envelope/plastic bag and sealed [21].

Samples were stored and handled according to manufacturers’ recommended storage conditions as described in the summary of product characteristics.

All sampled veterinary medicines were subjected to product information review (PIR). Sample screening testing and full quality control analysis were conducted at TMDA-World Health Organization (WHO) prequalified laboratory located in Dar es Salaam.

### Veterinary medicines selection criteria

Veterinary medicines that demonstrated poor performance in terms of quality in previously PMS programs and routine inspections or those reported to have a quality problem were targeted in this surveillance. These included mono-component: amprolium water-soluble powder, diminazene diaceturate powder for injection, levamisole injection, oxytetracycline 10% powder for oral and solution for injection, oxytetracycline 20% powder for oral and solution for injection, isometamidium hydrochloride powder for injection and albendazole suspension.

### Quality evaluation

#### Screening testing (Tier I)

Screening testing included PIR, visual inspection and identification test by Thin Layer Chromatography (TLC). Pharmacopeial monograph [30], in-house and/manufacturer methods were used wherever appropriate.

#### Product information review

Each collected sample was subjected to PIR. Parameters checked during PIR included but not limited to dosage form description, brand and generic names, strength, name and address of the manufacturer, batch or lot number, manufacturing and expiry date, TMDA registration number, packaging and pack size, availability and information on package information leaflet (PIL), information on the primary and secondary packaging, storage instructions, indications, warning instructions, language, and physical appearance of the primary and secondary package. Eligibility and correctness of the above information were checked against TMDA labelling and packaging guidelines and approved product information. The checked information was recorded on a standardized form.

**Visual inspection test** was conducted by examining the collected samples for discoloration, odour, leakage, excessive powder, powder caking, solubility, pH, and clarity and adherence to national legislation requirements on labelling.

**Product identification by thin-layer chromatograph** was used for product identification and semi—qualitative determination of active ingredients, related substances, and impurities present in the dosage forms. This method employed the principle of comparing spots test sample and reference solutions. The principal spot obtained with the test sample solution was required to correspond with the chromatographic runs of the standard solution in terms of colour, shape, size, intensity, and retardation factor (R_f_) value. The test sample was considered failed if the R_f_ value of the test sample was different by more than 10% from that of the standard sample and if the intensity of the spot was less than that of a reference containing 80% of the stated amount of the active pharmaceutical ingredient (API). This had to be observed in three independent experiments [21].

#### Full quality control testing (Tier-II)

All samples that failed the screening test, 10% of those which had passed the screening test, and those with doubtful results were subjected to full quality control testing [21]. Confirmatory testing was carried out at the TMDA-WHO prequalified laboratory as per pharmacopoeial monograph requirements [22] or manufacturer’s methods and/or in-house specifications [21]. Typical parameters tested were sterility, identification, assay, and related substances**.**

### Data management and statistical analysis

Inconsistencies was checked for all collected data and were then transferred to the SPSS software (version 23) for cleaning and analysis. Only dataset for PIR of the sampled veterinary medicines were analyzed using SPSS. Descriptive statistics was used to analyze continuous variables. Results are presented in form of frequencies and percentages.

## Results

### Veterinary medicines collected samples

A total of 238 selected veterinary medicines were collected between 2014 and 2017. The medicines were sampled from retail and wholesale pharmacies, veterinary clinics, and ADDO-vet in Tanzania Mainland. A large proportion of these samples were collected from private pharmacies 92% (220/238) and very few from veterinary clinics 1.3% (3/238).

In this study, 34.5% (82/238) of the sample were collected from Arusha, 31.1% (74/238) Shinyanga, and 0.8% (2/238) Kilimanjaro. Arusha and Shinyanga regions have the highest population of livestock when compared with Kilimanjaro region.

Furthermore, 24.3% (58/238) of all collected samples were those of oxytetracycline 10% solution for injection and powder for oral solution while 30.3% (72/238) were of oxytetracycline 20% solution for injection and powder for oral solution as highlighted in Table 1.

**Table 1:** Number of sampled veterinary medicines and subjected to full quality control analysis

Name of medicines	Samples collected and screened			Confirmatory test	
	**Samples collected**	**Samples screened and passed**	**Samples Failed**	**Samples eligible for confirmatory**	**Samples selected and tested**
Amprolium hydrochloride injection	14	14	0	2	14
Diminazene diaceturate powder for injection	31	31	0	3	31
Levamisole injection	12^a^	8	0	1	8
Albendazole solution	14^b^	12	0	1	12
Isometamidium chloride hydrochloride powder for injection	37	32	5	3	37
Oxytetracycline 10% powder for solution & solution for injection	58	58	0	6	58
Oxytetracycline 20% powder for solution & solution for injection	72^a^	71	0	7	71
**Total**	**238**	**226**	**5**	**30**	**231**

^a^ Indicates four (4) and one (1) sample of levamisole and oxytetracycline 20% water soluble powder respectively which expired before analysis

^b^ Indicates two (2) samples of albendazole suspension which were not registered and therefore not taken for laboratory analysis as it was not part of the program implementation

**Fig. 1:**
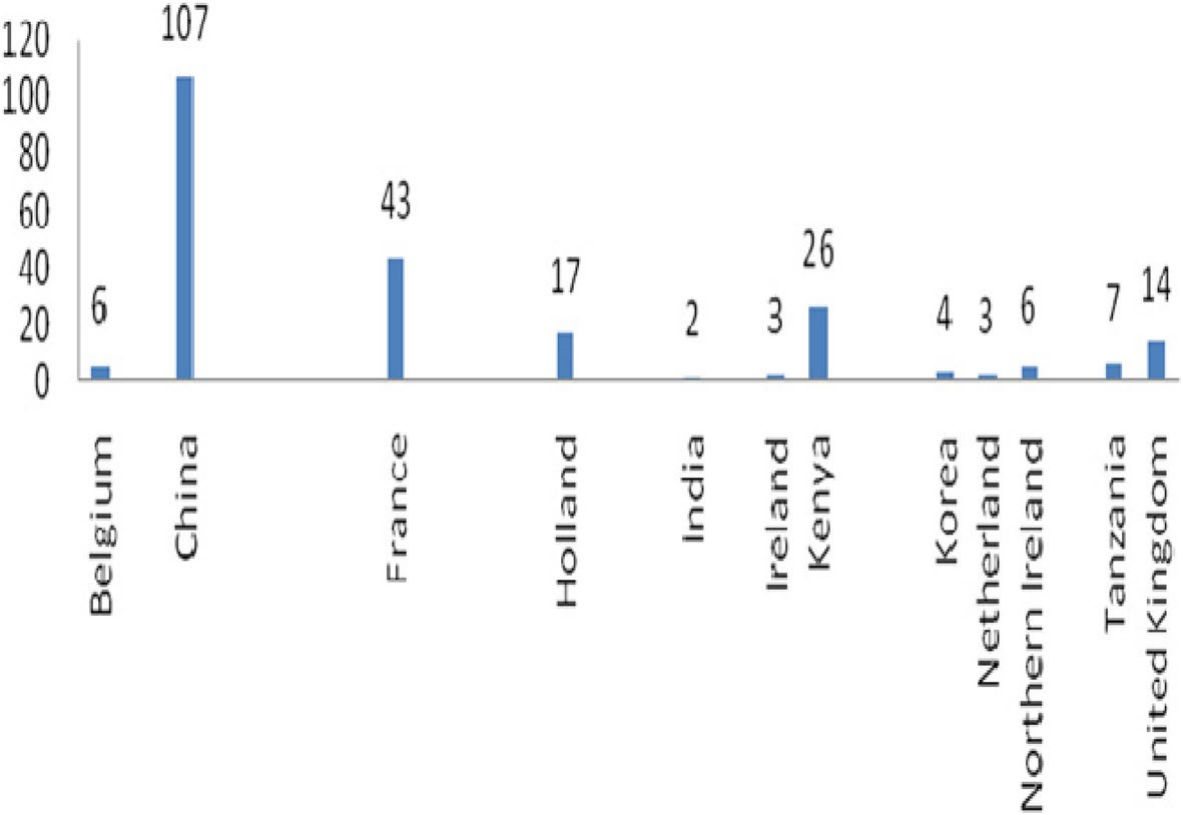
Number of samples of veterinary medicines imported and domestically manufactured

It was further observed that 97% (231/238) of the sampled veterinary medicines were imported, of which 47% (107/231) were from China and only 3% (7/238) were domestically manufactured as illustrated in Fig. 1.

### Laboratory tier I screening test

#### Product information review

**Fig. 2:**
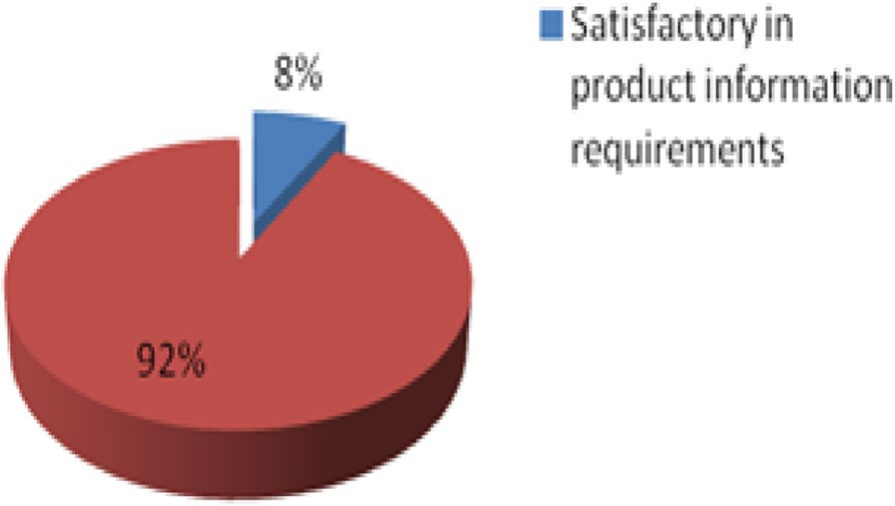
Conformity to product information requirements

All collected samples were subjected to PIR, only 8% (19/238) of the sampled veterinary medicines, complied with product information requirements as shown in Fig. 2.

**Fig. 3:**
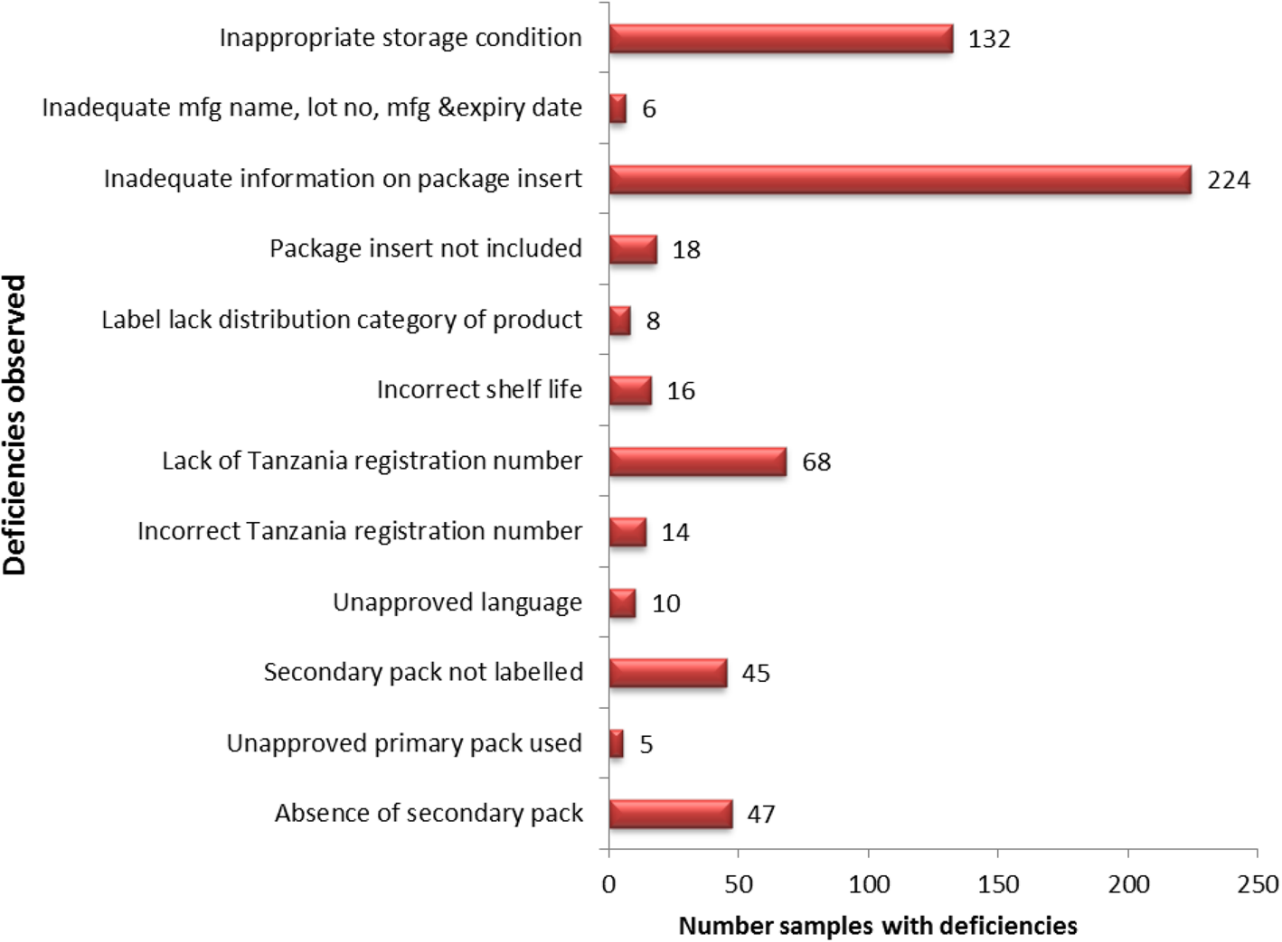
Deficiencies on the primary, secondary and package inserts

The most identified deficiencies were inadequate information on the package insert 94.1% (224/238), inappropriate storage conditions indicated 55.5% (132/238), lack of Tanzania registration number 28.6% (68/238), absence of secondary pack 19.7% (47/238) and secondary pack not labeled 18.9% (45/238). Of the sampled veterinary medicines two (2) of them were found not to be registered in Tanzania. All observed deficiencies for PIR are summarized in Fig. 3.

Again, in this survey, a total of 97.1% (231/238) samples, comprising 129 antibiotics, 68 trypanocides, 20 anthelmintics, and 14 anti-coccidia were subjected to visual inspection and identification tests. Even so, 3% (7/238) of samples could not be taken to the laboratory for analysis since two (2) were not registered and five (5) expired.

### Visual inspection test

All sampled veterinary medicines conformed to visual appearance requirements.

### Identification test

Of the 231 sampled veterinary medicines and subjected to identification testing, 97.8% (226/231) passed the identification test. The failed samples were of isometamidium hydrochloride powder for injection of 13.5% (5/37).

#### Full quality control testing (Tier-II)

A total of 25 samples out of the 231 (11%) subjected to full quality control, did not comply with the assay test requirement.

It was also noted that 13.5% (5/37) of the failed samples were isometamidium chloride hydrochloride powder for injection, 33.3% (4/12) albendazole solution, 29% (9/31) diminazene diaceturate powder for injection, 15.6% (5/32) oxytetracycline 20% solution for injection and 5.1% (2/39) oxytetracycline powder for solution.

The concentration of the API in those failed samples were observed to range between 72.8—122.8% compared to the acceptable limits of 95.0—105.0% for diminazene diaceturate powder for injection; 10.1—82.9% against 90.0—110.0% for albendazole solution; 0—84.1% against 95.0—102.0%; for isometamidium chloride hydrochloride powder for injection; 72.9 – 109% against 92.5—107.5% for oxytetracycline 20% solution for injection; and 20.8 – 116.0% compared to 90.0—110.0% for oxytetracycline 20% solution for injection.

## Discussion

The importance of PMS of veterinary medicines lies on the great overlap between human and animal medicines which have the same concerns over quality, safety, and efficacy. This calls for the one health approach on the quality, safety and efficacy of medicines as a strategy to fight against drug resistance. Above all, increased availability and demand for veterinary medicines is another driving factor.

The quality of veterinary medicines has been a major public health problem, particularly in developing countries [5, 13–18]. The content of API is the most important characteristic of a drug product as it determines treatment outcomes [5]. This study assessed the quality of selected 238 veterinary medicines sampled from various medicine distribution outlets in Tanzania Mainland.

Of the sampled veterinary medicines, 97% were imported from overseas, indicating the dependence of the country on importation. This observation is in agreement with a 2016 report on the low capacity of domestic pharmaceutical manufacturers in manufacturing human and veterinary medicines [23]. TMDA data of 2017 have indicated similar findings [24].

The sampled veterinary medicines were mostly from private pharmacies. This is of interest to law enforcement and adequate regulatory control on the availability of veterinary medicines largely in pharmacies as per TMDA requirements.

This study revealed that 11% of the tested medicines had a percentage content of APIwhich did not meet the official pharmacopoeial specifications [22] and/or manufacturer specification limits. Also, it was observed that 0.8% of the sampled medicines were not registered in Tanzania Mainland.

Results of the sampled medicines that failed the assay test correlates with study findings observed in Ethiopia, Kenya, Nigeria, South Korea, Cambodia, and Cameroon which averaged between22—69% [5, 13–18, 25–27]. Even so, similar or contrary findings for individual medicines had been reported [5, 18, 25, 28, 29]. In the Ethiopian study, 28% of the sampled and tested trypanocidal drugs (diminazene aceturate and isometamidium chloride hydrochloride) were substandard [18]. The findings of this study are in – line with the Ethiopian study that 20.5% of the surveyed trypanocidal medicinal products were substandard with the content of the claimed API ranging from 0—122.8%. A high prevalence (40%) of trypanocidal drugs with poor quality have been reported in the Togo study as well [28]. Low or no content of API of trypanocidal had been reported to cause major economic burden on livestock production such as increased animal deaths, retardation in growth and reproduction and drug resistance [14, 30, 31].

Of the sampled albendazole solution, 33.3% had a concentration below the official monograph specification of 90–110%, which is in agreement with findings from the Ethiopian study (30%) and Yemen (28.5%) studies [25, 29]. Nigeria study reported the presence of a high prevalence (55.5%) of albendazole tablets with poor quality in the legal market [16]. The non-compliance of the tested oxytetracycline 20% powder for injection in this study was (5.1%) which was similar to the results observed in the South Korean study 5% [5].

The use of **s**ubstandard veterinary medicines which contain API lower than the specified level may lead to treatment failure and ultimately animal deaths increased treatment costs for farmers due to repetitive treatment and development of drug resistance [9, 18, 25, 29]. In addition, the use of pharmaceutical products with a high concentration of claimed API is associated with drug toxicity and death.

Poor quality veterinary medicines identified by this surveillance were found in Tanzania Mainland legal market. A possible cause of availability of substandard veterinary medicines in Tanzania Mainland could be due to poor manufacturing processes, such as poor formulation, incorrect weighing, and mixing or intentionally done to reduce the cost of production [20, 32]. Another cause might be high temperature and humidity during storage and distribution processes leading to reduced product potency [33, 34]. Availability of porous borders could as well account for the same. The availability of such products in developing countries can be reduced through the strengthening of the national regulatory authorities [10]. lso, strengthening local pharmaceutical manufacturing industries through the provision of staff training, analytical equipment/laboratories and recruitment of adequately qualified manufacturing staff, adherence to quality assurance protocols and adequate regulatory control [10], might improve the availability of domestic manufacturers of veterinary medicines of good quality.

Compared with PIR results for human medicine categories in the implementation of PMS programs, results of the current study need attention and regulatory action (s) by the TMDA [11, 12, 35]. The most observed deficiencies were inadequate information on the product leaflets, inappropriate storage conditions and lack of Tanzania registration numbers.

Product leaflet is important source of information to pastoralists and farmers. Thus, inaccurate and uninformative labeling poses a potential risk to the health of livestock and humans such as the development of antimicrobial drug resistance, drug toxicity, treatment failure and death [36].

Product registration ensures that veterinary medicines circulating in the Tanzania market are safe, effective, and of good quality. Since some unregistered veterinary medicines were found circulating in the market; this calls for more vigilant and frequent inspections at ports of entry. The challenge of having porous borders also needs to be addressed [13].

All pharmaceutical products must be stored under conditions provided by manufacturers to ensure that their potency and quality are not compromised during the distribution process and storage. In this study, it was observed that some of the manufacturers did not indicate proper storage conditions whilst some provided conditions that are not achievable in tropical climates, such as “store below 25 °C”. In a country like Tanzania which falls under zone IVb climatic conditions, it is not easy to achieve/maintain the aforementioned storage conditions [37]. Improper storage of pharmaceutical products negatively affects potency, quality, efficacy, safety of the pharmaceutical products and subsequently compromises the quality of life of the end-users [33, 34]. Therefore, proper storage conditions must be maintained throughout the product life cycle as indicated in the TMDA registration guidelines.

## Conclusion

Veterinary medicines of poor quality were found circulating in the legal markets of Tanzania Mainland. Poor compliance of manufacturers of veterinary medicines to product information requirements was very prominent. Therefore, continuous post marketing surveillance is pivotal in ensuring that veterinary medicines of good quality are circulating in the Tanzania Mainland market.

## Data Availability

All relevant data generated and analyzed during this study are available from the Director General of TMDA; email: adam.fimbo@tmda.go. tz on reasonable request.

## Abbreviations

Vet-ADDO: Veterinary Accredited Drug Dispensing Outlet
MSD: Medical Stores Department
TMDA: Tanzania Medicines and Medical Devices Authority
PMS: Post Marketing Surveillance Program
WHO: World Health Organization
